# Descriptive versus causal morphology: gynandromorphism and intersexuality

**DOI:** 10.1007/s12064-023-00385-1

**Published:** 2023-01-12

**Authors:** Giuseppe Fusco, Alessandro Minelli

**Affiliations:** Department of Biology, University of Padova, Padua, Italy

**Keywords:** Sex anomalies, Sex determination, Sexual differentiation, Sexual development, Gonochorism, Hermaphroditism

## Abstract

In animal species with separate sexes, abnormal individuals with a mix of phenotypically male and phenotypically female body parts are generally indicated as gynandromorphs, whereas individuals with intermediate sexual phenotypic traits are generally indicated as intersexes. However, this distinction, clear as it may seem, is neither universally agreed upon, nor free of critical issues. In consideration of the role of sex anomalies in understanding normal development, we reassess these phenomena of abnormal sexual development, taking into consideration the more recent advances in the study of sex determination and sexual differentiation. We argue that a distinction between gynandromorphism and intersexuality, although useful for descriptive purposes, is not always possible or sensible. We discuss the conceptual and terminological intricacies of the literature on this subject and provide reasons for largely, although not strictly, preferring a terminology based on descriptive rather than causal morphology, that is, on the observed phenotypic patterns rather on the causal process behind them.

## Introduction

According to Ford (2012), the oldest published account of a zoological specimen with an abnormal combination of male and female features is the description by Nicholls (1730) of a lobster dissected and analysed for the Royal Society. In the original paper, this animal was described as hermaphrodite, whereas Ford (2012), who recognizes it as a bilateral gynandromorph, does not hesitate to call it also an intersex. The occurrence of these three terms (hermaphrodite, gynandromorph and intersex) in past or recent descriptions of one and the same specimen is an iconic example of long-standing difficulties in classifying sex anomalies.

In modern usage, as a first approximation, abnormal individuals of gonochoric (separate-sex) species with a mix of phenotypically male and phenotypically female body parts are generally indicated as *gynandromorphs*, whereas individuals with intermediate sexual phenotypic traits are generally indicated as *intersexes*. On the contrary, the term *hermaphrodite*, or *monoecious* in botanical terminology, generally indicates a normal individual that at the same time or in different stages of its life can produce both male and female gametes (Fusco and Minelli 2019). Hermaphroditism is the normal sex condition in many seed plants and some groups of invertebrates. The use of the term to indicate sex anomalies in separate-sex organisms, including humans, is discouraged (Dreger 2005).

In this paper, we will reassess the phenomena of abnormal sexual development described under the headings of gynandromorphism and intersexuality in animals, taking into consideration the more recent advances in the study of sex determination and sexual differentiation. Although we will deal almost exclusively with sex anomalies in gonochoric animals, when the study of sexually anomalous specimens focusses on the reproductive system, the gonads especially, we cannot ignore cases that could be described as to verge towards hermaphroditism, a question to which we will return towards the end of the article.

Most examples we will cite are about arthropods, the clade we know better, but our discussion is not limited to sex anomalies in this group. We will argue that a distinction between gynandromorphism and intersexuality can be very useful for descriptive purposes, especially when simply based on the phenotypic appearance of the anomaly. However, we will also show that, in consideration of the complexity of the mechanisms of sex determination and sexual differentiation (and their interactions), a distinction is not always possible or sensible.

Since there is no general term in use to include both gynandromorphism and intersexuality, when these are intended as distinct classes of phenomena or phenotypes, in the present article, we will use the umbrella term *sex anomaly* to refer to both and to some other anomalies in sexual phenotype not covered by either term.

Following, in part, Richter and Wirkner’s (2014) identification of four main approaches to morphology, intended as description, functional analysis, comparison and explanation of an organism’s parts, we will frame our discussion on gynandromorphism and intersexuality in terms of both descriptive and causal morphology. Based on an overview of these sex anomalies in terms of their phenotypic patterns and possible (or putative) causal explanations, we will discuss the conceptual and terminological intricacies of the literature on this subject and provide some reasons for largely, although not strictly, preferring a terminology based on descriptive rather than causal morphology.

## The inconsistent terminology for sex anomalies

To our knowledge, the oldest use of the term ‘gynandromorphism’ is the following telegraphic account (Newport 1845: 373): “Amongst the subjects of physiological interest exhibited at our meetings [of the Entomological Society], I may notice an instance of Gynandromorphism in *Arctia Caja,* by Mr. Evans”. No description or illustration accompanied this sentence, but we can easily accept that that specimen of the garden tiger moth (Lepidoptera: Erebidae) exhibited a mosaic of phenotypically male and phenotypically female body parts, perhaps in the wings.

In the second half of the nineteenth century, the term was extensively applied to similarly anomalous specimens, insects especially. It was only in 1915 that Richard Goldschmidt, who had previously described as gynandromorphs all sexually anomalous phenotypes obtained till that date through crosses between individuals of different races of the gypsy moth (*Lymantria dispar*; also in Erebidae), that he realized the need for a new term. Rather than being mosaics of phenotypically male and phenotypically female parts, his moths “represent[ed] a quantitatively determined intermediate level between the two sexes. If we were to designate a female as 0 and a male as 100, then a certain of my bred animals represents level 3 or 21 or 75, etc.; so not a mixture of both sexes, but a certain point between the two extremes female-male. […] So it seems to me necessary to introduce a different designation for the phenomenon dealt with here. In the future I will refer to the sexual intermediate stages as intersexes and speak of male or female intersexes, depending on whether they are males on the way to femininity or females on the way to masculinity: the appearance itself would then be called intersexuality” (Goldschmidt 1915: 566; our translation).

For a while, this distinction proved to be adequate, and terminology was temporarily stabilized, but it was eventually challenged by the expansion of knowledge on sexual development and the diversification of research approaches in different fields of studies, from transmission and developmental genetics, to mutagenesis and zooculture.

The whole history of these phenomena and associated terms reveals a long-standing and ever-increasing overlap between strictly descriptive morphology and causal morphology. This is complicated by the mostly hypothetical nature of causal explanations and the concurrence of multiple, mechanistically different causes in the production of similar morphological patterns. At any rate, a diversity of processes potentially responsible for the production of the morphologically anomalous sexual phenotypes must be expected, due to the diversity and complexity of mechanisms involved both in the primary establishment of sex and in the translation of the latter into sexual differentiation of the whole individual and its different parts.

If Cline (1984: 235) still accepted that “true intersexes [are] intermediate in phenotype between males and females even at the level of individual cells”, in a classic book on *Intersexuality in the Animal Kingdom* Reinboth (1975) had already suggested that because of the increased knowledge of “the bewildering variability present in the sexual organization of members of the animal kingdom”, Goldschmidt’s (1915) original meaning of the term intersexuality must be abandoned. Eventually, the term is used by some authors as inclusive (e.g. Ford 2012) or synonym (e.g. Arnold et al. 2013) of gynandromorphism and even broadened to simply mean the presence, in a single individual, of both male and female characteristics or of intermediate sexual characteristics (Atz 1964), that is, used for any kind of anomaly in sexual phenotype (e.g. Traut et al 2008; Short et al. 2014; Chandler et al. 2018), often involving genital ambiguity, and unusual combinations of karyotype and sexual phenotype (Allen 2009). Finally, with a strictly gonadal perspective, Grilo and Rosa (2017: 717) define intersexes as “individuals of gonochoristic species possessing oocytes or distinct stages of spermatogonia, at varying degrees of development, within the normal gonad of the opposite gender (i.e. spermatocytes in the ovary or oocytes in the testis)”, thus limiting intersexuality to cases that could be dubbed as ‘rudimentary hermaphroditism’, because of the presence of a bifunctional mixed gonad (Grilo and Rosa describe it as an ovotestis, thus using the term by which the bisexual gonad of a number of hermaphrodites, including many gastropods, is currently described).

The inconsistent use of the two terms in the literature is evident, and later in this article, we will discuss the possibility to put some order in the matter. However, the principal aim of the present contribution is not the formulation of a normative proposal to solve a semantic question, but rather to show the diversity of the phenomena of sex anomalies, considering limits and advantages of different descriptive and classificatory choices. Before delving into a discussion of sex anomaly phenomena and the logical organization of knowledge about them, we will briefly review the most common sex anomaly patterns, along with their most frequent causes. For convenience of description, a distinction between gynandromorphism and intersexuality based on the pattern of the anomaly is adopted.

## Patterns of sex anomaly

**Fig. 1:**
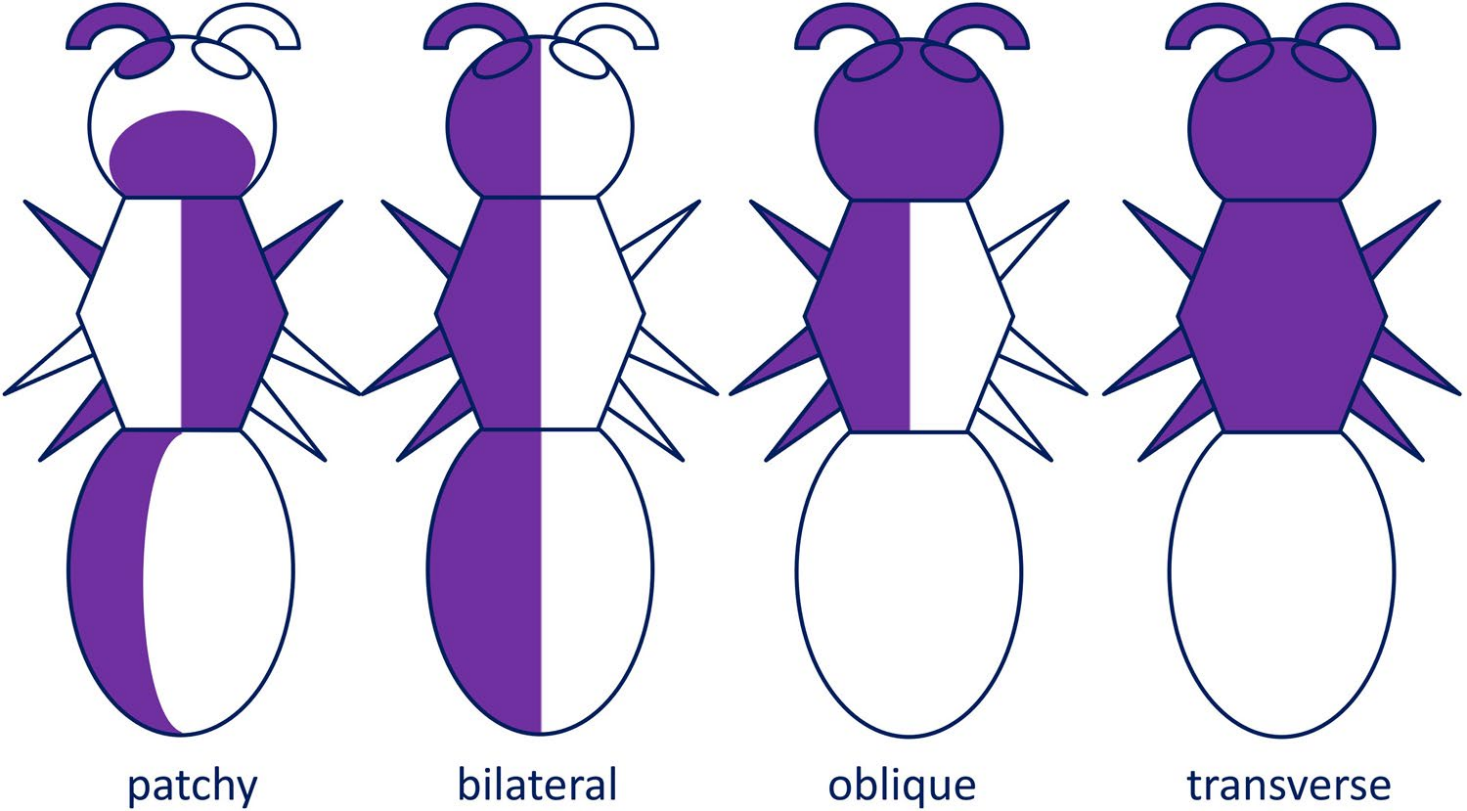
Schematics of the most common patterns of symmetric gynandromorphism. Colours indicate the opposite-sex (male vs. female) phenotype of different body parts

In several gonochoric animals, individuals with a mix of phenotypically male and phenotypically female anatomical parts may occur in nature or can be induced experimentally. These individuals are generally indicated as gynandromorphs. In some of them, male-type tissues and female-type tissues produce a patchwork throughout the body, but often occur in a pattern with some kind of symmetry. In *bilateral gynandromorphs*, one side of the body has male characters, the other side female. In *transverse gynandromorphs* (also known as *polar gynandromorphs*), a plane transversal to the main body axis separates male and female body parts. Finally, in *oblique gynandromorphs*, the boundary between the different-sex body parts crosses the sagittal plane diagonally (Fig. 1).

Many cases of gynandromorphism have been described, mostly among insects, chelicerates (spiders and mites), decapod crustaceans, and birds.

In several gonochoric animals, only partly coinciding with those where gynandromorphs occasionally occur, abnormal individuals can present body parts with an intermediate sexual phenotype. These are generally indicated as intersexes and are relatively common in some groups of crustaceans, especially among isopods and amphipods. Intersex features can be limited to a set of characters or structures. For example, in the milkweed bug *Oncopeltus fasciatus*, even specimens with dramatic intersex genitalia still display a sternite shape typical of their presumptive sex (Just et al. 2021). In addition, the term intersex has been extended to include individuals with unambiguous sexual phenotype (either male or female) that is opposite to their genetic sex (Narita et al. 2010).

Patterns of phenotypic expression of an intersexual condition are more taxon-specific that those in gynandromorphism, which can be classified based on pure symmetry. Description of patterns of intersexuality comprises the specification of the sexually dimorphic anatomical structures (including genitalia) and body organs (including gonads) affected by the anomaly. For instance, in the isopod *Armadillidium vulgare,* four principal types of intersexes are described in Legrand and Juchault (2006): (1) functional males with female genital orifices; (2) individuals in which the gonad is a functional ovary provided with a vestigial testicular vesicle and a likewise rudimentary vas deferens; (3) female individuals that closely resemble the previous category and that can only be distinguished from these at a physiological level; and (4) males the gonad of which is a testis provided with an oviduct. Different combinations and numbers of genital openings distinguish different intersex types in the crayfish *Cherax quadricarinatus* (Parnes et al. 2003; Levy et al. 2020).

## Causes of sex anomaly

Before reviewing the most common causes of these sex anomalies, let’s first distinguish between *sex-determination systems* and *sex-determination mechanisms* (Fusco and Minelli 2019).

Systems of sex determination are traditionally classified based on the nature of the primary causative agent in the specification of an individual’s sex. These can be qualified with a variable level of detail, so that, for instance, one can distinguish between *genetic* and *environmental* sex-determination systems, or more specifically describe an XY system among the former, or a *temperature-dependent* system among the latter.

The mechanisms of sex determination are the developmental processes that interpret these first signals, and different mechanisms can be associated with the same sex-determination system. For instance, with the XY chromosomal system, the sex of an individual can be determined by a mechanism that depends on the presence of a Y chromosome (mammals) or, alternatively, by a mechanism through which the sex correlates to the number of X chromosomes (*Drosophila*). Mechanisms of sex determination gradually blend into the developmental *processes of sexual differentiation* that implement the attainment of a given sex condition (e.g. Gilbert and Barresi 2016).

Sex anomalies can have multiple causes. To classify these causes, different options are open, which different authors have adopted based on convenience or the peculiarities of the cases they were studying. This multiplicity of alternative classifications stems from the very nature of the phenomenon we are discussing. Both sex determination and sexual differentiation are complex process, often including many regulative steps; thus, one can see causes at different levels: there can be more *remote/ultimate causes*, like hybridization or parasitic infection, and more *proximate causes*, like the missing expression of a key sex-determining gene, and these are obviously not mutually exclusive.

Without the aim of suggesting a new classification, but only with the objective of showing the causal disparity of these phenomena, we survey here the causes of sex anomalies, using, again for convenience, the usually accepted distinction between gynandromorphism and intersexuality.

### Causes of gynandromorphism

In gynandromorphs, different tissues express different sex identities. This occurs more frequently in organisms where sex determination and/or sexual differentiation is not exclusively controlled at a systemic level, allowing some independence in sexual differentiation, at least in some body regions. This is, for instance, the case of insects and other arthropods, but also birds and nematodes, where sexual differentiation is largely a cell-autonomous process (Bachtrog et al. 2014). Conversely, sexual differentiation is largely dependent on circulating sex hormones in most vertebrates (other than birds) and crustaceans, where it depends on gonad-dependent endocrine regulation (vertebrates), or the products of sex-specific endocrine gland (crustaceans) (Toyota et al. 2021). Although less frequent, gynandromorphism can still occur in these groups, for instance, deriving from a differential hormone sensitivity of different organs or body parts.

#### Genetic mosaicism

Genetic mosaicism is the condition of a multicellular individual carrying different genomes that originated from the genome of a single founder cell (Fusco and Minelli 2019). In organisms with a chromosomal sex-determination system (e.g. most insects), gynandromorphism can result from genetic mosaicism at the level of sex chromosomes, while in organism with haplodiploid sex-determination system (e.g. hymenopterans), it can result from genetic mosaicism that involves the ploidy level (i.e. the number of homologous sets of chromosomes). Heterogeneity in the chromosomal complement can originate during cleavage mitoses in embryogenesis. For instance, the loss of an X chromosome in a cell of a *Drosophila* embryo with XX karyotype will produce a mix of X0 (male) and XX (female) cells. Note that it is the mechanism of sex determination that establishes whether a given chromosomal mutation will lead to a different sex specification. For instance, among the XY systems in dipterans, in *Drosophila,* the initial switch is associated with the ratio between the number of X chromosomes and the number of homologous sets of autosomes (X:A ratio system; Erickson and Quintero 2007), while in the mosquito *Aedes aegypti*, sex determination depends on the presence of the Y chromosome (dominant-Y system; Hall et al. 2015; Blackmon et al. 2017).

Another possibility of producing a genetic mosaic is through fertilization of a binucleate egg (Narita et al. 2010). Binucleate eggs can occur when the second polar body is not discarded during female gametogenesis. In *Drosophila* and in *Bombyx*, double fertilization (simultaneous fertilization by two sperm cells) of a binucleate egg can produce an individual with female and male parts (with XX and XY karyotype in *Drosophila* and with ZW and ZZ karyotype in *Bombyx*, respectively). Similarly, in *Apis* (haplodiploid sex-determination system), the fertilization by one sperm cell of a binucleate egg can result in an individual with male (haploid) and female (diploid) body parts. When the polar body is a cell on its own, rather than a supplementary nucleus in the egg cell, the result of a double-fertilization event should be more properly termed a *genetic chimera*, rather than mosaic (a chimera is a multicellular individual made of cell populations originating from more than one founder cell; (Fusco and Minelli 2019). Chimeras with discordant sex karyotype have been reported in several organisms, including humans (Madan 2020).

A genetic mosaic can also result from parasite infection. A delay in the diploidization caused by the endocellular parasitic bacterium *Wolbachia*, occurring after the first cleavage, can produce a mix of diploid (female) and haploid (male) cells in the parasitic wasp *Trichogramma* (Stouthamer and Kazmer 1994).

#### Epigenetic sex mosaicism

In organisms with either genetic or environmental sex determination, mosaicism can affect the expression of genes (or their alternative splicing variants) downstream of the first sex-determining signal, that are part of the developmental pathway of sexual differentiation. Even here, the details of this option crucially depend on the specific mechanism of sex determination. For instance, in Hymenoptera, cell lineage-specific male sex determination can derive from defects in the maintenance of the inductive signal of the *fem* gene, so that it fails to mediate its own synthesis. In the honeybee, gynandromorphic phenotypes have been obtained in the lab by RNAi-induced knockdown of *fem* (Gempe et al. 2009), while Sommaggio et al. (2021) have made a case for an epigenetic origin of a recurrent gynandromorphic pattern in natural populations of *Megachile* wild bees.

#### Gynandromorphism in systems with hormone-dependent sexual differentiation

In organisms with sex hormones, gynandromorphism can be produced by genetic or epigenetic defects at the level of hormone-coding genes (or their expression), hormone receptors, or other elements of the cascade of sexual differentiation pathways.

Sexual differentiation in crustaceans occurs under the influence of a circulating male hormone, produced by an androgenic gland. Impaired production and/or circulation of the hormone can yield spectacular cases of bilateral gynandromorphism (Legrand and Juchault 2006), as recorded in a range of crustacean species, for instance, among lobsters (Chace and Moore 1959) and crabs (Micheli 1991).

Differential sensitivity of different tissues (somatic and/or gonadic) to the concentration of sex hormones or other sex-signals, or a biased distribution of these molecules, explain also some rare forms of gynandromorphism recorded among vertebrates with gonad-dependent endocrine sexual differentiation, for instance, among snakes (Krohmer 1989) and rodents (Hollander et al. 1956).

### Causes of intersexuality

In intersexes some or all tissues of an individual express intermediate sex identity. Restriction of intersexual phenotype to certain body parts is easier if sex determination and/or sexual differentiation is not controlled at a systemic level, and however, this is not strictly necessary.

#### Chromosomal mutations

Depending on the sex-determination mechanism, some chromosomal mutations (aneuploidy) affecting sex chromosomes, or accidents at syngamy, can produce a non-standard chromosomal arrangement. For instance, cells of a *Drosophila* triploid intersex with XXY/AAA karyotype differentiate into an intermediate sexual phenotype. Bridges (1921) observed in these intersexes considerable phenotypic variation, from more-female to more-male types, involving several dimorphic characters, like the presence of sex-combs on the tarsi of the fore legs (a male character), but also the gonads and the external genitalia.

#### Genetic or epigenetic alteration of downstream sex-determining genes

Intersexes can result from crosses between different strains of the same species or between closely related species (Narita et al. 2010). In the moth *Lymantria dispar*, crosses between different geographic strains result in the generation of intersexes that exhibit a uniformly intermediate phenotype (Goldschmidt 1934; but see below, "Reasons for not stressing a distinction between gynandromorphism and intersexuality"). Since these individuals have female karyotype (ZW), the cause of the intersexual phenotype is considered to be the incomplete masculinization of genetic females due to a particular allelic combination that may affect the normal expression of the sex-determining genes. This unbalanced condition of interracial hybrids is not unlikely in consideration of the fact that the sex-determining systems of arthropods tend to evolve and diverge quite rapidly (Beukeboom and Perrin 2014).

#### Developmental causes

Alteration of the developmental environment can interfere with the normal pathways of sex determination and/or sexual differentiation, resulting in intersex phenotypes. This includes the effects of factors like temperature, photoperiod or anthropogenic pollutants, but also those of symbionts or parasite infection.

High temperatures can induce partial feminization of genetic males in the mosquito *Culex stimulans*, but masculinization of genetic females in the bagworm *Dahlica triquetrella* (Narita et al. 2010, and references therein, sub *Solenobia triquetrella*). Intersex and other reproductive abnormalities associated with environmental pollution have been observed in a number of crustacean species, for example, in the amphipod *Echinogammarus marinus* (Ford et al. 2006) and in several species of copepods (Moore and Stevenson 1991, 1994).

Maternally inherited endosymbionts, known as reproductive parasites, have evolved strategies to convert non-transmitting male hosts into transmitting females through feminization of prospective males and induction of parthenogenesis (Fusco and Minelli 2019). In arthropods, bacteria of the genus *Wolbachia* can cause the feminization of males, which in species with heterochromosomes can result in individuals with a female phenotype despite their male karyotype, as reported for the butterfly *Eurema hecabe* (Narita et al. 2007) and the isopod *Armadillidium vulgare* (Cordaux et al. 2011). Parasites other than microorganisms, for example, among the flatworms (e.g. Christensen and Kannerworff 1965) and the nematodes (e.g. Kahanpää 2008) can affect the development of reproductive organs and/or secondary sexual characters in various arthropods as well (Baudoin 1975).

## Descriptive versus causal morphology

Up to now, the distinction between gynandromorphism and intersexuality has been based on either the causes of the anomaly, or the observed pattern (phenotypic appearance) of the anomaly, that is, in terms of either causal or descriptive morphology (sensu Richter and Wirkner 2014; see Introduction). Here, we describe the rationale behind each of the two options, along with possible conceptual and/or operational drawbacks in the formulation and/or application of the different criteria (Table 1).

**Table 1:** Summary chart of the different criteria adopted to distinguish gynandromorphs from intersexes, along with the rationale behind each option and the possible conceptual and/or operational drawbacks

	Diagnosis	Rationale	Drawbacks
Distinction based on causes	Gynandromorphs are genetic mosaic Intersexes are genetically uniform individuals	Apparently based on a hardwired (genetic) feature of the organism	Does not apply to organisms with environmental sex determination Can be applied only when genetic uniformity has been probed Does not provide a comprehensive categorization of the genetic causes of the observed patterns Requires further qualification according to categories that indicate the alternative morphological outcomes
Distinction based on pattern (body parts)	Gynandromorphs present anatomical parts with male characters and parts with female characters Intersexes have one or more anatomical parts of intermediate sex type	Apparently of straightforward applicability even in the absence of adequate knowledge on underlying processes	Intersexuals at one level of description may be qualified as gynandromorphs at a more detailed level Structures of intermediate sex type can be qualified as such also for the presence of different, topographically overlapping features of different sex type A structure can have features that qualifies it neither as intermediate between the two sex types, nor as male or female
Distinction based on pattern (characters)	Gynandromorphs presents characters with male sex states along with characters with female sex state Intersexes have one or more characters of intermediate sex state		Does not apply to qualitative characters Distinction depends on the level of detail in the description Some character states are neither male or female, nor intermediate

### Distinction based on causes

**Fig. 2:**
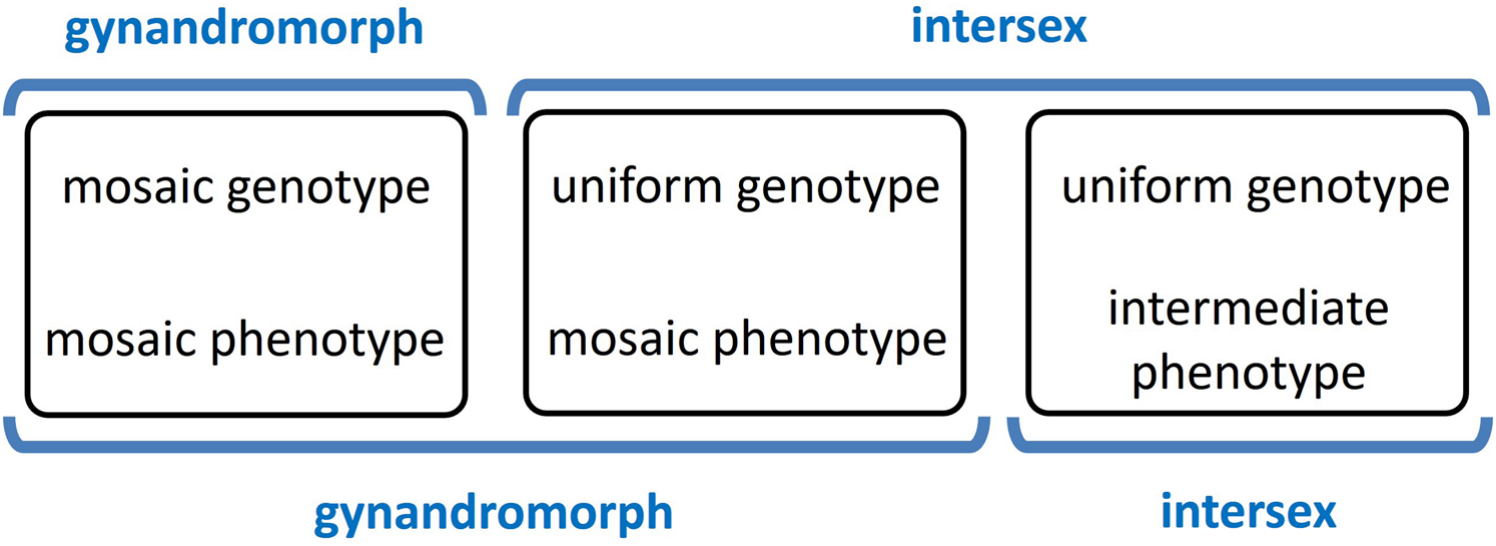
Schematics of the most widely adopted distinctions between gynandromorphism and intersexuality. A classification based on causal morphology, here the genotypic constitution of the organism (above), is contrasted with a classification based on descriptive morphology, here the phenotypic appearance of the organism (below)

Most frequently, gynandromorphs are defined as genetic mosaics of genetically pure male and pure female tissues, while intersexes are defined as genetically uniform individuals, their intermediate sexual appearance being the result of anomalous sexual differentiation under the influence of an abnormal genotype or karyotype or of abnormal developmental conditions (Stern 1968; Narita et al. 2010). Depending on the specific mechanisms of sex determination and sexual differentiation, an intersex could appear as a phenotypic mosaic of male and female parts or as a uniform phenotype of intermediate sex state (Fig. 2).

This distinction, although apparently based on a hardwired feature of the organism, presents several shortcomings:

1. The distinction has no biological significance if applied to organisms with environmental sex determination. However, individuals with a mix of male and female features or with intermediate sex features occur also in organisms where sex is not genetically determined, as, for instance, in the natural populations of the amphipod crustacean *Gammarus duebeni*, where the sex-determining environmental clue is the photoperiod to which the animal is exposed in an early, sensitive phase of development (Dunn et al. 1996)
2. An observed specimen cannot be qualified until its genetic uniformity is probed and ascertained, and in most cases, this will remain unknown
3. At a closer inspection, such an alleged process-based distinction is actually a classification based merely on the individual’s genetic constitution. However, this is only one of the possible early clues for sexual differentiation, and signals downstream of these first clues are equally potential locations of defects producing anomalies in sexual development. Actually, genes at the top of the hierarchy of regulatory interactions between sex determination and sexual differentiation are more evolutionary labile than downstream target genes (Marin and Baker 1998). Therefore, a distinction between gynandromorphism and intersexuality based on genetic uniformity does not appear to provide a satisfactory comprehensive categorization of the disturbed processes that may produce the observed patterns
4. It does not eliminate the need for terms to indicate the overall morphological result of the abnormal developmental process. For example, Narita et al. (2010) use the terms “phenotypically mosaic” and “phenotypically uniform” to indicate alternative morphological outcomes.

### Distinction based on pattern

Authors who adopted a distinction between gynandromorphs and intersexes based on the phenotypic pattern of the anomaly have mostly accepted Goldschmidt’s (1915) definitions: gynandromorphs present anatomical parts with male characters and parts with female characters, whereas intersexes present a uniformly intermediate sexual phenotype (Fig. 2). Although at a first glance this distinction may appear very clear and of straightforward applicability, this is not so.

For a deeper analysis, it is sensible to distinguish between a description of the phenotypic pattern based on *anatomical body parts*, e.g. legs, antennae, external genitalia, and a description based on *phenotypic characters* like body size, number of antennal segments, density of setae. Note that characters are not necessarily referable or restricted to a given body part (i.e. they can be an attribute of the phenotype that applies to more than one body part), and that a single structure can exhibit more than one sexually dimorphic character. We will analyse these two alternatives in turn.

#### Body part-based pattern

An individual with a mosaic of male- and female-type structures would be a gynandromorph, while an individual with one or more body parts of intermediate sex type would be an intersex. However,

1. The division of the body into structural units is largely arbitrary. A structure made of substructures of different sex type could be classified as of intermediate sex type (thus, the individual would be an intersex), but dividing the same structure into smaller units, each of strictly male or female sex type, intersexuality would vanish into gynandromorphism. Intersexuals at one level of description may be gynandromorphs at another level. For instance, in sexually anomalous individuals of the wild bee *Megachile perihirta*, the front legs are of intermediate sex type; however, in detail, the femora are coloured as in male, the tibiae are entirely dark as in female, the proximal half of the front tarsi is enlarged and whitish as in male, and the apical part of the tarsi is narrowed and black as in female (Mitchell 1929; Sommaggio et al. 2021)
2. A structure can be qualified as of intermediate sex type for two very different reasons: either (i) the structure is intermediate between the normal male and female conditions, or (ii) different, topographically overlapping features (e.g. the number of antennal segments vs. the density, length or colour of setae covering them) are of different sex type. An example of (i) is the WZ intersexes of the gypsy moth, *Lymantria dispar*, where the wings are uniform in colour and can take different tonalities from female-like light brown, to medium, to male-like dark brown (Mosbacher 1973, 1975). An example of (ii) is the antennae of sexually anomalous specimens of the wild bee *Megachile angelarum*, where the number of flagellomeres (10) is of the female type, whereas the shape of the flagellomeres (distinctly longer than wide) is of the male type (Mitchell 1941; Sommaggio et al. 2021)
3. A structure can have features that are not intermediate between the two sex types, but different from both. For instance, the pollen-collecting apparatus in *Megachile angelarum* gynandromorphs is neither of the female type nor absent as in males, but of unique phenotype (Mitchell 1941; Sommaggio et al. 2021).

#### Character-based pattern

In the common language of comparative morphology, here used in an intraspecific context, a *morphological character* is any observable feature, or trait, in an organism, while the particular form or value that the character manifests in a given specimen is its *state* (Sokal and Sneath 1963). Accordingly, an individual with a mosaic of male and female character states would be a gynandromorph, while an individual with one or more characters of intermediate sex state would be an intersex. There are deficiencies in this choice as well:

1. An intersex can be qualified only for quantitative characters (either metric or meristic), since the concept of intermediate state does not apply to characters treated (or coded) as qualitative
2. The definition of a character and its alternative states is arbitrary. Different choices in describing the same phenotype may lead to qualifying a sexually anomalous individual as either a gynandromorph or an intersex. Coarse-grained descriptions will more easily result in recording it as an intersex, while very detailed descriptions will more easily depict it as a gynandromorph. The first option may be suggested by the individual variation in the phenotypic expression of a sex anomaly in the face of apparently similar causes, as in case of the *doublesex* mutants in *Drosophila* (Hildreth 1965). The second option is generally preferred for naturally occurring cases where the focus is on the disparity in the composition of male and female features among sexually anomalous specimens of the same species or population (e.g. Sommaggio et al. 2021)
3. As in the case of a description based on body parts, the recorded anomalous character state is sometimes other than intermediate between the male and female states. In the case of quantitative characters, its value may be outside the interval of values delimited by the joint distribution of the two sexes. For qualitative characters, this distinctive state may have an otherwise unrecorded phenotype. For instance, *Drosophila* triploid intersexes tend to have larger body size than flies of either sex and distinctive roughish eyes (Bridges 1921).

### Reasons for not stressing a distinction between gynandromorphism and intersexuality

As noted by other authors (e.g. Agnew 1979; Nihei and Carvalho 2002), even when a clear criterion to distinguish between gynandromorphism and intersexuality is stated, there are often difficulties in assigning a specific case to one class or the other. Once more, an example is offered by *Lymantria* hybrids. In a late revisitation of their phenotypic status, Goldschmidt (1949); see also (Mosbacher 1973, 1975) remarked that whereas in more female-like intersexes (except for those derived from crosses involving the so-called Gifu race as one parent) even single-cell structures like the sensilla of the antennae and the wing scales were intermediate between the male and female phenotypes, the wings of more male-like intersexes were instead a mosaic of female and male parts. Indeed, even in his 1915 paper, where he introduced the distinction between intersexuality and gynandromorphism, Goldschmidt had admitted that the hybrids’ wings “at certain levels reveal sharply defined parts of female or male colouring” (Goldschmidt 1915: 566; our translation). Gynandromorphic and intersexual patterns coexisting in the same specimen have been recorded in field-collected arthropods, e.g. in the linyphiid spiders *Entelecara flavipes* and *Micrargus herbigradus* (Roberts and Parker 1973).

Also, beyond the practical issue of the availability of all the necessary information for a correct classification, a distinction is not always possible in principle. It would be an easy exercise in descriptive biology to show that, by tuning the level of the description, many reports of intersex phenotypes could be reformulated in terms of gynandromorphism, and vice versa.

Finally, stressing a dichotomy between gynandromorphism and intersexuality hides the fact that there are sex anomalies that do not fit either morphological category. These are, for example, the characters or structures seen above, that have a phenotype that is neither intermediate nor of the opposite sex with respect to other features. There are also anomalies that cannot even be described as an abnormal expression of a character of one of the two sexes. In gastropod molluscans, *imposexuality* (the superimposition of male characters onto the female) derives from the development of additional, non-functional male sexual organs (i.e. vas deferens and/or penis) by females of gonochoric species (Barroso et al. 2000; De Wolf et al. 2001). Not to mention those gonadal anomalies, interpretable as rudimentary forms of hermaphroditism, that would place specimens outside the framework, strictly limited to gonochoric animals, within which gynandromorphism and intersexuality were first defined, as in the case of a recently found specimen of the Pacific spadenose shark (*Scoliodon macrorhynchos*), which presented reproductive tracts of both sexes and ovotestes containing viable male and female gametes (Zhao et al. 2017).

## Conclusions

Studies in sex determination and sexual differentiation are growing fast, revealing new molecular details on how sex identity develops, and their increasingly extensive taxonomic coverage is showing unexpected patterns in the evolution of these organismal features. The study of the gene-regulatory networks involved in sex-determination is disclosing how these different mechanisms work, what factors are implicated, upstream and downstream of the genetic cascades, and how they are regulated to bring about the observed plasticity (Herpin and Schartl 2015). At the same time, in an evolutionary perspective, the study of the same networks has generated multiple scenarios and hypotheses on the evolution and the evolvability of the same mechanisms, to explain which evolutionary forces could support such transitions and turnovers (Beukeboom and Perrin 2014).

The contribution of phenotypic anomalies, either naturally occurring or experimentally induced, to our understanding of normal development does not need to be stressed here, and those affecting the development of sexual characters and sexual identity are no exception (Germani et al. 2018; Held 2021). We think that in these studies, a critical choice of the terminology, accompanied by apt explanations or qualifications, is conductive to a better understanding of the phenomena under scrutiny. Both an exceedingly liberal nomenclature and, on the opposite, exceedingly rigid terminological definitions can generate so called ‘conceptual traps’, i.e. descriptive terms that can hamper further investigation and understanding (Fusco 2008).

We have shown that a classification of sex anomalies based on sex-determination or sexual-differentiation processes is weakened by the fact that the same sex anomaly can have multiple causes, even within the same individual, and that a distinction based on patterns is not always possible even in principle, critically depending on our level of description. In synthesis, there are no definitions of intersexuality and gynandromorphism that could be considered biologically significant in all the possible combinations of causes and effects involved in these sex anomalies.

All in all, among the two options for a distinction between gynandromorphism and intersexuality based on descriptive or causal morphology, respectively, we advocate the former. This avoids the complications of tracing it back to causes, which in complex, multi-level systems, like those of sex determination and sexual differentiation, are generally hard to identify and isolate. Instead, a pattern-based distinction, possibly making explicit the level of the description, allows the use of a consistent terminology even in the absence of adequate knowledge on the underlying genetic, epigenetic and developmental process involved. This choice also helps to avoid stressing the distinction between the two phenomena too strongly. Nonetheless, a degree of caution should regularly accompany the use of terms such as ‘gynandromorphism’ and ‘intersexuality’.
